# Developmental Outcomes of Preterm and Low Birth Weight Toddlers and Term Peers in Rwanda

**DOI:** 10.5334/aogh.2629

**Published:** 2019-12-17

**Authors:** Alain Ahishakiye, Marie Claire Abimana, Kathryn Beck, Ann C. Miller, Theresa S. Betancourt, Hema Magge, Christine Mutaganzwa, Catherine M. Kirk

**Affiliations:** 1 Rwinkwavu District Hospital, Ministry of Health, Rwinkwavu, RW; 2 Partners In Health/Inshuti Mu Buzima, Rwinkwavu, RW; 3 Division of Global Health and Social Medicine, Harvard Medical School, Boston, US; 4 School of Social Work, Boston College, Boston, US; 5 Division of General Pediatrics, Boston Children’s Hospital, Boston, US; 6 Institute of Health Care Improvement, Addis Ababa, ET

## Abstract

**Background:**

As neonatal care improves in low-resource settings, more preterm or low birth weight (LBW) babies are surviving, but little is known about their long-term outcomes. Globally, preterm and/or LBW babies are at increased risk of mortality, malnutrition, and developmental delay.

**Objectives:**

We aim to describe the differences in development in rural Rwandan children at 24–36 months of age born preterm and/or LBW compared to their peers born term or normal birth weight (term/NBW), and to assess factors associated with poor development.

**Methods:**

We conducted a cross-sectional study using secondary data analysis from two combined datasets from 2014, using Ages and Stages Questionnaire (ASQ-3) for developmental assessment and anthropometrics for nutritional status (stunting and wasting). Demographic and clinical factors associated with poor developmental outcomes in univariate regression at α = 0.20 were included in a full model; we used backward stepwise penalized multivariable logistic regression to identify a final model at α = 0.05.

**Findings:**

In total, 445 children were included; 405 term/NBW, and 40 preterm and/or LBW. Half of them (n = 234; 52.6%) had developmental delay, including 207 (51.1%) among term/NBW and 27 (67.5%) among preterm and/or LBW (p = 0.048). In the final model, term/NBW children with stunting alone had a significant increase in the odds of developmental delay (OR 2.05, 95% CI 1.37–3.07), and children with wasting had a borderline statistically significant increased odds of developmental delay (OR 5.79, 95% CI 0.98-34.39). Being preterm and/or LBW and not stunted completely predicted delay.

**Conclusion:**

Half of the children had developmental delay in our sample from rural Rwanda. Preterm and/or LBW infants were more likely to have developmental delay, and the main predictor of developmental delay was stunting, with high rates of stunting observed also in term/NBW infants. Interventions to reduce undernutrition and prevent prematurity and LBW, alongside investments to promote early stimulation for optimal development, are needed if gains in addressing developmental delay are to be made.

## Introduction

Among children under five years in low- and middle-income countries (LMICs), an estimated 250 million (44%) are at risk of not reaching their full developmental potential [1,2]. A child’s developmental potential is highly influenced by individual factors including nutritional status and perinatal risk factors such as prematurity, in addition to the conditions of the home environment such as exposure to stimulation and chronic poverty [3]. The early years of life are a critical period of development as a brain’s neural pathways develop most rapidly and proliferate from conception to age two [4]. Stressors during this period including prematurity, undernutrition, trauma or stress, contribute to long-term effects on the brain’s structure [5]. In countries in which the prevalence of risk factors for poor childhood development outcomes such as malnutrition and nutrient deficiency are high, higher rates of disability and developmental delay are often observed [6].

Children born with risk factors for developmental delay such as prematurity are a high-risk group [7,8] whose outcomes in LMICs are not well understood [9]. Fifteen million babies worldwide are born preterm each year, with more than 80% of them in Africa and south Asia [10]. In total, nearly a quarter of infants born in sub-Saharan Africa in 2015 had low birth weight (LBW) [11]. The combined issues of prematurity, LBW and small for gestational age (SGA) are difficult to differentiate in LMICs where precise information on gestational age is often unavailable [12]. Even though healthcare in resource-limited countries is improving, infants born preterm and LBW are still at high risk of poor outcomes, such as death [8,13,14]; and for those who survive, nutritional deficiencies and developmental challenges [14,15]. Kerstjens et al. found that both moderate and early preterm infants had more frequent problems with fine motor, communication, and personal-social functioning compared with full-term infants [16]. Prematurity and LBW contribute to intellectual disability and neurodevelopmental deficits leading to infant and childhood morbidity, particularly intellectual and learning disabilities [17,18].

Rwanda has worked to reduce neonatal mortality through newborn survival initiatives, including a National Neonatal Care Protocol [19], and the establishment of neonatal care units (NCUs) in every public hospital to care for sick and small newborns [20]. Recent data from Rwanda estimate that 6% of newborns are LBW (birth weight less than 2.5 kg) and 10% are preterm [21,22]. Through the efforts to improve care for sick and small newborns, more preterm and/or LBW babies are surviving into childhood, but little is known about their long-term outcomes. The Rwanda Ministry of Health (MOH) has invested in Pediatric Developmental Clinics (PDCs) for the developmental and nutritional follow-up of high-risk babies, including those born preterm and LBW [23].

Kirk et al. found poor health, nutrition and development outcomes among children born preterm and LBW when they were ages 1 to 3 years in rural Rwanda [15]. To our knowledge, there is no study in Rwanda comparing nutritional and developmental outcomes in early childhood of infants born with prematurity and LBW (preterm and/or LBW) to those infants born at normal gestational age and normal birthweight (term/NBW). In this study, we aim to extend knowledge by describing and assessing the differences in development at 24 to 36 months of age between these groups and to assess factors associated with poor development in preterm and/or LBW and term/NBW toddler-age children in rural Rwanda.

## Methods

### Study setting, design and data collection

We conducted a cross-sectional analysis, using secondary data from two studies conducted in rural communities across 11 of 30 districts in Rwanda. Data from the original two datasets were combined and are described below.

The first study collected data in 2014 on a random sample of children aged 0–11 and 24–36 months located in rural communities in 10 districts that were pre-selected based on high rates of stunting and poverty to receive the implementation of the Early Childhood Development and Family (ECD&F) intervention by Imbuto Foundation and UNICEF Rwanda (“UNICEF study”) [24]. The data were collected through the collaboration of the Harvard T.H. Chan School of Public Health, University of Rwanda, and Partners In Health/Inshuti Mu Buzima (PIH/IMB) from August to November 2014 to establish a baseline of children’s development prior to ECD&F program implementation. Potential participants were identified through the enumeration of households using community health workers (CHWs) registers, as CHWs are responsible for tracking all children under age five in Rwandan villages [25]. Following enumeration of age-eligible children, a random sample of children was identified using a random number generator in Microsoft Excel; these sampled households were then visited for household data collection resulting in a total sample of 884 children. More detailed methods are described elsewhere [24].

The second study collected data from hospital registers of infants born preterm and/or LBW who were discharged alive from the Neonatal Care Unit at Rwinkwavu District Hospital (RDH) between October 2011 and October 2013 and who received routine community-based care (“Preterm/LBW study”) [25]. RDH is a Ministry of Health hospital located in Kayonza District that is supported by PIH/IMB. Children were excluded if they had congenital malformations, hypoxic ischemic encephalopathy, or were not preterm and/or LBW. Using geographic and family information from the hospital records, household data were collected on children alive in November to December 2014, when the children were between 1–3 years (n = 86). More detailed methods are described elsewhere [15].

In the combined dataset for this study, we included all children from both databases aged 24–36 months at the time of assessment. We excluded children missing developmental status data which is the primary outcome of the study.

The same data collector teams and nutrition and development assessment tools were utilized to collect household data in both studies. Data were merged into one file for analysis; any child with status of preterm and/or LBW was included in the preterm and/or LBW group, whether the child was from the UNICEF study or the preterm/LBW study.

### Definitions

Our main outcome, “developmental delay”, was defined using Ages and Stages Questionnaire version 3 (ASQ-3), a parent-reported developmental screening instrument that has been successfully used in Rwanda for providing estimates of the proportion of children whose development may be of concern [15,24,26]. The ASQ-3 forms are specific to the child’s age, with 21 total forms ranging from 2 months to 60 months (with corrections up to 24 months for prematurity) which ask the caregiver to report on 30 questions based on the child’s age. We defined “developmental delay” as a child whose ASQ-3 score fell below cut-points for their age in any one of five ASQ-3 domains, including gross motor, fine motor, communication, problem solving and personal-social [27].

We defined “preterm” as a dichotomous variable; babies born before 37 weeks of gestational age were defined as preterm [28]. Babies born with a weight under 2.5kg were defined as “LBW” [29]. In our study, children with either preterm or LBW were defined as “preterm and/or LBW”. We defined children born at term and with normal birth weight as “term/NBW” [28]. In the UNICEF study, children whose caregivers reported that they were born LBW or premature were categorized as preterm and/or LBW for analysis.

Nutritional status was collected using standard anthropometric procedures and cut-offs from World Health Organization Growth Standards [30]. “Stunting” was defined as height/length for age z-score < –2 standard deviations (SDs) below the mean, “wasting” defined as weight-for-height/length age z-score < –2 SDs, and “underweight” defined as low weight-for-age z-score < –2 SDs. Stunting and wasting were considered “severe” if z-scores were less than 3 SDs below the mean. Mid-Upper Arm Circumference (MUAC) measurements, which is a proxy measure of nutrient reserve in muscles and fat, were reported as a continuous variable [30].

“Ubudehe” is a community-based ranking of relative wealth used in Rwandan national statistics. Ubudehe, at the time of study, was categorized into 6 rankings, with 1 being lowest wealth status and 6 being highest wealth status [31].

We observed an interaction between premature and/or LBW and stunting with respect to developmental delay, so we reported these factors as a combined 4-item categorical variable: “preterm and/or LBW and stunting” equals 0 if neither premature and/or LBW nor stunted, 1 if “not premature and/or LBW but stunted”, 2 if “preterm and/or LBW but not stunted”, and 3 if “both premature and/or LBW and stunted”.

### Data analysis

We described patient demographics, clinical characteristics, nutritional status and developmental status using frequencies and percentages for categorical data and median and interquartile ranges for continuous data. We reported the proportion of poor developmental outcomes with 95% confidence intervals with a precision of 0.2. We assessed the relationship between patient demographics, clinical characteristics, nutritional status outcomes (stunted, underweight, wasted) and developmental (developmental delay, no delay) using Chi-squared test or Fisher’s Exact test for categorical variables and Wilcoxon rank sum or Kruskal Wallis tests for continuous variables.

We used logistic regression to identify factors associated with poor developmental outcomes built using backward stepwise procedures for factors associated at α = 0.20 in bivariate analyses. Because we observed small numbers in some cells, we used penalized likelihood logistic regression for the final model to provide robust standard errors, confidence intervals and p-values [32]. All factors significant at the α = 0.05 significance level were retained in the final model. The data were analyzed using Stata v.15.1 (Stata Corp, College Station, TX, USA).

### Ethics

The preterm/LBW study received approval from the National Health Research Committee, Rwanda National Ethics Committee (RNEC), the Rwanda Ministry of Health, and the Boston Children’s Hospital Institutional Review Board (IRB). The UNICEF study received approval from RNEC and the Harvard T.H. Chan School of Public Health IRB. Because this is a secondary analysis of deidentified data, additional ethics review was not required; technical review was provided by the PIH/IMB research committee.

## Results

**Figure 1:**
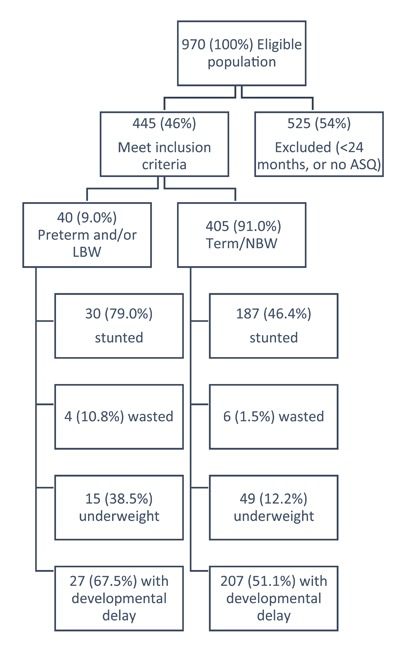
Flow diagram of children eligible for this study, and their nutritional and developmental characteristics, from two datasets of (1) a random sample of children born term/normal birth weight (“UNICEF study”) who were aged 24 to 36 months when data was collected in 2014; and (2) children born preterm and/or low birthweight (“Preterm/LBW study”) who were discharged from the neonatal care unit (NCU) at Rwinkwavu District Hospital (RDH) between October 2011 and October 2013 and were aged 24 to 36 months when data was collected in 2014.

Among 970 eligible children in the combined dataset, 445 (46%) met inclusion criteria of ages 24–36 months, of which 40 (9.0%) were born preterm and/or LBW and 405 (91.0%) were born term/NBW (Figure 1). We observed more females than males (55.5%), and 23 (57.5%) preterm and/or LBW and 224 (55.3%) term/NBW were females (Table 1). Of 441 children with nutritional status recorded, 217 (49.2%) had stunting, 10 (2.3%) had wasting, and 64 (14.5%) were underweight. Among 40 children born preterm and/or LBW, 30 (79.0%) had stunting, 4 (10.8%) had wasting and 15 (38.5%) were underweight compared to 187 (46.4%) with stunting, 6 (1.5%) with wasting and 49 (12.2%) underweight children born term/NBW. Based on ASQ-3 scores, 234 (52.6%) children had developmental delay. Twenty-seven (67.5%) children born preterm and/or LBW and 207 (51.1%) children born term/NBW had developmental delay according to the ASQ-3.

**Table 1:** Sociodemographic and clinical characteristics of infants born preterm and/or low birth weight (“Preterm/LBW study”) and infants born at term or normal birth weight (NBW) (“UNICEF study”) in Rwanda, at 24–36 months in 2014; N = 445 unless otherwise stated.

	Born preterm and/or LBW		Born term/NBW	

	N = 40		N = 405	

	n	%	n	%

**Child age (months)**				
24–26	10	25.0	82	20.3
27–29	8	20.0	123	30.4
30–32	14	35.0	102	25.2
33–36	8	20.0	98	24.2
**Child sex**				
Male	17	42.5	181	44.7
Female	23	57.5	224	55.3
**Province**				
East	38	95.0	82	20.3
Kigali	0	0.0	71	17.5
North	0	0.0	83	20.5
South	1	2.5	83	20.5
West	1	2.5	86	21.2
**Caregiver’s relationship, N = 437**				
Mother	38	95.0	373	94.0
Father	0	0.0	13	3.3
Other	2	5.0	11	2.8
**Caregiver’s education, N = 424**				
None completed	10	25.0	65	16.9
Primary completed	26	65.0	294	76.6
Secondary or higher completed	4	10.0	25	6.5
**Caregiver’s marital status, N = 394**				
Not married	0	0.0	65	18.4
Married	16	40.0	226	63.8
Living with partner (not married)	17	42.5	63	17.8
Divorced/widowed	7	17.5	0	0.0
**Household wealth (Ubudehe categories)**				
Extremely poor	0	0.0	16	4.0
Moderately poor	9	22.5	86	21.2
Poor	23	57.5	206	50.9
Not poor	0	0.0	1	0.3
Unknown or N/A	8	20.0	96	23.7
**Other children in household**				
No other children in household	8	20.0	84	20.7
1–3 other children in household	21	52.5	257	63.5
4 or more other children in household	11	27.5	64	15.8
**MUAC (mm) [mean, SD], N = 442**	143.5	11.6	150	11.7
**Stunted, N = 441**				
Normal (z score ≥ –2 SD)	8	21.1	216	53.6
Moderate (z score < –2 SD)	30	79.0	187	46.4
**Wasted, N = 436**				
Normal (z score ≥ –2 SD)	33	89.2	393	98.5
Moderate (z score < –2 SD)	4	10.8	6	1.5
**Underweight, N = 440**				
Normal (z score ≥ –2 SD)	24	61.5	352	87.8
Moderate (z score < –2 SD)	15	38.5	49	12.2
**ASQ-3 development status**				
On-track	13	32.5	198	48.9
Developmental delay	27	67.5	207	51.1

In bivariate analysis (Table 2) factors associated with developmental delay at an alpha of 0.20 included age of child at assessment (p = 0.087), child sex (p = 0.121), premature birth (p = 0.048), stunting (p = 0.002), wasting (p = 0.018) and underweight (p = 0.003). These factors were included in our full model.

**Table 2:** Factors associated with developmental delay on ASQ-3 in infants born preterm and/or low birth weight (“Preterm/LBW study”) and infants born at term/normal birth weight (NBW) (“UNICEF study”) in Rwanda, at 24–36 months in 2014; N = 445.

	ASQ Delay, N = 234		ASQ No delay, N = 211		p-value

	n	%	n	%

**Child age (months)**					0.087
24–26	58	63.0	34	37.0	
27–29	70	53.4	61	46.6	
30–32	53	45.7	63	54.3	
33–36	53	50.0	53	50.0	
**Child sex**					0.121
Male	96	48.5	102	51.5	
Female	138	55.9	109	44.1	
**Province**					0.210
East	70	58.3	50	41.7	
Kigali	39	54.9	32	45.1	
North	43	51.8	40	48.2	
South	35	41.7	49	58.3	
West	47	54.0	40	46.0	
**Caregiver’s relationship, N = 437**					0.288
Mother	215	52.3	196	47.7	
Father	5	38.5	8	61.5	
Other	9	69.2	4	30.8	
**Caregiver’s education, N = 424**					0.691
None completed	42	56.0	33	44.0	
Primary completed	163	50.9	157	49.1	
Secondary or higher completed	16	55.2	13	44.8	
**Caregiver’s marital status, N = 394**					0.464
Not married	36	55.4	29	44.6	
Married	118	48.8	124	51.2	
Living with partner (not married)	44	55.0	36	45.0	
Divorced/widowed	5	71.4	2	28.6	
**Household wealth (Ubudehe categories)**					0.368
Extremely poor	11	68.8	5	31.3	
Moderately poor	45	47.4	50	52.6	
Poor	120	52.4	109	47.6	
Not poor	0	0.0	1	100.0	
Ubudehe unknown or N/A	58	55.8	46	44.2	
**Other children in household**					0.827
No other children in household	51	55.4	41	44.6	
1–3 other children in household	144	51.8	134	48.2	
4 and more children in household	39	52.0	36	48.0	
**Prematurity and/or low birth weight**					0.048
Yes	27	67.5	13	32.5	
No	207	51.1	198	48.9	
**MUAC (mean, SD)**	148.0	11.6	151.0	11.8	0.007
**Stunted, N = 441**					0.002
Normal (z score ≥ –2 SD)	102	45.5	122	54.5	
Moderate (z score < –2 SD)	131	60.4	86	39.6	
**Wasted, N = 436**					0.018
Normal (z score ≥ –2 SD)	222	52.1	204	47.9	
Moderate (z score < –2 SD)	9	90.0	1	10.0	
**Underweight, N= 440**					0.003
Normal (z score ≥ –2 SD)	188	50.0	188	50.0	
Moderate (z score < –2 SD)	45	70.3	19	29.7	

Factors associated with developmental delay in the final model included child age and stunting and history of prematurity and/or LBW, and wasting (Table 3). Older age at assessment was associated with a reduced odds of developmental delay; this was only statistically significant for the children aged 30 to 32 months compared to children in the youngest age group (24–26 months) (OR 0.55, 95% CI 0.31–0.99). Having stunting or a history of preterm and/or LBW was associated with higher odds of developmental delay in all categories compared to children with neither concern. Children with stunting but no history of prematurity and/or LBW had double the odds of developmental delay (OR 2.05, 95% CI 1.37–3.07). Children who had both histories of preterm and/or LBW and stunting had increased odds of delay compared to normal birthweight non-stunted peers, but this was not statistically significant (OR 1.68, 95% CI 0.75–3.78). Children with wasting had borderline statistically significant higher odds of developmental delay (OR 5.79, 95% CI 0.98–34.39).

**Table 3:** Multivariable logistic regression model with odds ratios (OR), p-value and confidence intervals (CI) for developmental delay as measured by ASQ.

	ASQ Screen for Developmental Delay					

	Full			Adjusted		

	OR	95% CI	p-value	OR	95% CI	p-value

**Child age (months)**						
24–26	Ref	Ref	Ref	Ref	Ref	Ref
27–29	0.71	[0.41–1.26]	0.244	0.71	[0.40–1.24]	0.229
30–32	0.57	[0.32–1.01]	0.055	0.55	[0.31–0.99]	0.045
33–36	0.63	[0.35–1.15]	0.134	0.61	[0.34–1.10]	0.098
**Child sex**						
Male	Ref	Ref	Ref			
Female	1.36	[0.91–2.02]	0.132			
**Interaction: prematurity and/or low birth weight (LBW) and stunting**						
Not preterm and/or LBW and not stunted	Ref	Ref	Ref			
Not preterm and/or LBW but stunted	1.89	[1.23–2.91]	0.004	2.05	[1.37–3.07]	<0.001
Preterm and/or LBW but not stunted	15.93	[0.87–290.44]	0.062	16.13	[0.89–291.97]	0.060
Preterm and/or LBW and stunted	1.38	[0.60–3.21]	0.451	1.68	[0.75–3.78]	0.206
**MUAC (mean, SD)**	0.99	[0.98–1.01]	0.522			
**Wasted**						
Normal (z score ≥ –2 SD)	Ref	Ref	Ref	Ref	Ref	Ref
Moderate (z score < –2 SD)	4.10	[0.65–25.91]	0.134	5.79	[0.98–34.39]	0.053
**Underweight**						
Normal (z score ≥ –2 SD)	Ref	Ref	Ref			
Moderate (z score < –2 SD)	1.33	[0.67–2.66]	0.415			

## Discussion

We found high rates of developmental delay in our study population (52.6%), and significantly more developmental delay among children born preterm and/or LBW (67.5%) compared to term/NBW born children (51.1%) at age 2–3 years. However, we also found the high prevalence of stunting in our population (49.2%) as the major driver of developmental delay in children ages 24–36 months.

While we expected preterm and/or LBW children to have a higher prevalence of developmental delay [14,15] compared to term/NBW children, we also found high rates of developmental delay among term/NBW children. We found that compared to non-stunted term babies, stunted children without history of preterm and/or LBW had a significantly higher prevalence of development delay, independent of other factors. Small size at birth is one of the leading predictors of stunting globally [33], and thus the high rate of growth faltering and subsequent developmental delay even among children without this perinatal risk factor in our sample is notable. This finding is similar to findings from Ethiopia that children born with intrauterine growth restriction and children born normal birth weight showed similar anthropometric outcomes (underweight and stunting) by age two [34]. The subsequent strong association between stunting and developmental delay is well known. A meta-analysis conducted in 15 LMICs showed that severe stunting was negatively associated with overall development across all countries [35]. Similarly, a study in sub-Saharan African countries showed that among the determinants of developmental delay studied, stunting was the strongest predictor of developmental delay [36].

We found significantly higher rates of developmental delay at 2–3 years of age among infants born preterm and/or LBW. This increase in developmental delay was also seen in neighboring Burundi, where 27% of LBW children had some form of developmental impairment based on a screening questionnaire at age two after discharge from a neonatal care unit [37]. In Malawi, infants born preterm were more likely to screen positive for disability on a parent-reported assessment tool and had higher rates of developmental delay at 18 months compared to term infants (22.8% versus 10.9%) [38]. Similar high prevalence of developmental delay has been shown in other sub-Saharan African countries, including Ghana and Central African Republic, where 24% and 51%, respectively, of children in the general population aged 2–4 years, screened positive for disability [39]. These rates are also dramatically higher than the rates of delay seen in high-income countries [16] and higher than we expected given that most children born premature are expected to have caught up developmentally to their term peers by age two [40]. However, the disparity across settings is not surprising given that a baby born in a high-income country before 32 weeks’ gestational age has twice the chance of surviving the neonatal period without later neurodevelopmental impairment than if that baby is born in South Asia or sub-Saharan Africa [41].

We expected rates of stunting to be high in our study, given the high prevalence of stunting in rural Rwanda (41%) [21] and the eastern African region (26%–57.7%)[42], however, the 42.9% prevalence of stunting observed in our study exceeds the WHO “very high” threshold for stunting [43], and deserves particular attention. Rates of stunting were especially high in children with a history of preterm and/or LBW (79%) and higher than the national average among term/NBW children as well. While the high prevalence of stunting among preterm and/or LBW children is not unexpected, this finding is alarming and consistent with other findings from this region. Van den Boogaard et al. found an 80% prevalence of stunting among infants born LBW in Burundi, compared to a 50% prevalence nationally [37]. Stunting and prematurity/LBW are closely linked: in a meta-analysis by Christian et al. it was found that increased odds of childhood stunting was associated with prematurity, SGA, and LBW [44]. Globally, SGA at birth is one of the biggest risk factors for stunting in early childhood [33]. However, accurately capturing SGA is difficult in LMIC contexts where accurate information on gestational age is often unknown [12], which is why in our study we were not able to assess SGA given limitations in precision of gestational age measurements. Because all of our children with preterm and/or LBW who were not stunted had developmental delay, we were also unable to look at these variables separately.

The prevalence of wasting among the children with a history of term/NBW (1.5%) was similar to that in Rwanda (2%) [21], but more than five times the national average among the preterm and/or LBW children (10.8%) in our study. Among all children in the study who were wasted, there was a borderline significant association with developmental delay. Exclusive breastfeeding, timely introduction and adequacy of complementary feeding, and access to effective treatment of wasting are needed for all children, but with particular emphasis on those infants born preterm and/or LBW to reduce developmental delay which could reduce the prevalence of severe wasting by 61.4%, and could also decrease malnutrition-related deaths [45].

While being preterm and/or LBW was significantly associated with delay in bivariate analysis, it was not a statistically significant independent predictor of poor developmental outcomes in the final model. We suspect that the lack of significance was due to our small sample size of children categorized as preterm and/or LBW, and very high prevalence of stunting, rather than a finding actually contrary to most published literature. All of the children with preterm and/or LBW who were not stunted had developmental delay per the ASQ-3. A 2017 study assessing factors associated with developmental delay among the preterm and/or LBW study population found that children who were SGA and very LBW (birth weight less than 1.5kg) had worse developmental scores than children who were born at a normal size for gestational age or at higher weight [15]. However, in our study, term/NBW children were also found to have surprisingly high rates of stunting and developmental delay. Together, these findings suggest that interventions to reduce stunting and malnutrition, as well as those to prevent prematurity and LBW will be of benefit in addressing developmental delay. Targeted health and nutrition interventions are needed during the preconception period, pregnancy, lactation and the first 2 years of life, also known as the first 1,000 days [46,47], to prevent preterm and/or LBW, stunting and to improve outcomes for children in the early months and years of life. There is strong global evidence for complementary interventions that are also needed to support children across Rwanda to promote positive parenting and early stimulation to support children to achieve their optimal development [3]. Some of these interventions include: preconception education to promote parenting skills for child development promotion, provision of complementary foods support, ensuring neighborhoods are clean and safe, and access to health and educational services [3]. In Rwanda, some promising models are emerging such as *Sugira Muryango* (Strengthen the Family)—a home-visiting intervention with a successfully completed pilot [48]. Projects that can be brought to scale will be needed to improve outcomes for children across Rwanda, and special attention should be paid to those born preterm and/or LBW throughout their early years.

Our study has several limitations. First, there are some differences between the specific indicators collected by the UNICEF dataset of term/NBW infants and the preterm and/or LBW dataset, and so only those indicators available in both datasets are included in this study. However, the data available for the two databases is sufficiently similar to allow comparison of key socio-demographic characteristics; our primary outcomes of child development and nutritional status were measured using the same tools and methods, and the data were collected by the same study team. Second, there is no tool for developmental evaluation that has been developed specifically for Rwandan children, and so estimates of developmental delay in our study are based on standard Western cut-points on the ASQ-3. However, the ASQ-3 has been adapted and translated into Kinyarwanda, the primary language spoken in all regions of Rwanda, and is used widely in sub-Saharan Africa. Third, there were few children in the study with prematurity and LBW; a small sample size likely affected our ability to detect risk differences between our groups. Variables such as prematurity and caregiver education that are relevant in larger studies might be less so in this study due to small sample size. Larger studies in this higher-risk population would be of great benefit to understanding risks specific to Rwanda that may be applicable to other LMICs. In the UNICEF study, mothers may have been unable to report LBW or prematurity accurately due to lack of available information. However, previous studies in similar settings have found no significant difference in parental reporting of birth size with hospital records 2–3 years after birth [49]. Further, mothers were better able to report size at birth when she had a facility-based delivery [50], which is 91% of all births in Rwanda [21]. Therefore, we are confident of maternal recall of size at birth and any errors in recall of birth weight most likely would have attenuated our findings. Lastly, precise estimation of gestational age in Rwanda is a major challenge, as is the case in other LMICs, therefore we are not able to determine the difference between preterm and appropriate weight for gestational age from newborns who had intrauterine growth restriction.

## Conclusions

The alarmingly high rates of developmental delay and stunting among toddlers in the study of both children with a history of preterm and/or LBW and those born term/NBW warrants attention to the nutritional and developmental needs for these children, and country-level strategies that prioritize prevention of prematurity and intrauterine growth restriction as part of stunting reduction efforts. Since stunting and wasting were both independently associated with developmental delay, a special focus of resources on developmental interventions to support children with malnutrition, and children born preterm and/or LBW who are more likely to be malnourished, would be warranted. The interaction between prematurity and/or LBW and stunting with developmental delay demonstrates the close linkages between these factors and strategies, and interventions should focus on all children at risk for these conditions especially for children in poor, rural communities.
